# Endogenous Retroelement Activation is Implicated in Interferon‐α Production and Anti–Cyclic Citrullinated Peptide Autoantibody Generation in Early Rheumatoid Arthritis

**DOI:** 10.1002/art.43083

**Published:** 2025-01-27

**Authors:** Faye A. H. Cooles, Gemma Vidal Pedrola, Najib Naamane, Arthur G. Pratt, Ben Barron‐Millar, Amy E. Anderson, Catharien M. U. Hilkens, John Casement, Vincent Bondet, Darragh Duffy, Fan Zhang, Ruchi Shukla, John D. Isaacs

**Affiliations:** ^1^ Newcastle University Newcastle upon Tyne United Kingdom; ^2^ Newcastle University and Newcastle Hospitals NHS Foundation Trust Newcastle upon Tyne United Kingdom; ^3^ Institut Pasteur, Université Paris Cité Paris France; ^4^ Harvard Medical School Boston Massachusetts; ^5^ Newcastle University and Northumbria University Newcastle upon Tyne United Kingdom

## Abstract

**Objective:**

Endogenous retroelements (EREs) stimulate type 1 interferon (IFN‐I) production but have not been explored as potential interferonogenic triggers in rheumatoid arthritis (RA). We investigated ERE expression in early RA (eRA), a period in which IFN‐I levels are increased.

**Methods:**

ERE expression (long terminal repeat [LTR] 5, long interspersed nuclear element 1 [LINE‐1], and short interspersed nuclear element [SINE]) in disease‐modifying treatment‐naïve eRA whole‐blood and bulk synovial tissue samples was examined by reverse transcription–polymerase chain reaction and NanoString alongside IFN‐α activity. Circulating lymphocyte subsets, including B cell subsets, from patients with eRA and early psoriatic arthritis (ePsA) were flow cytometrically sorted and similarly examined. Existing established RA and osteoarthritis (OA) synovial single‐cell sequencing data were reinterrogated to identify repeat elements, and associations were explored.

**Results:**

There was significant coexpression of all ERE classes and *IFNA* in eRA synovial tissue samples (n = 22, *P* < 0.0001) and significant positive associations between whole‐blood LINE‐1 expression (n = 56) and circulating IFN‐α protein (*P* = 0.018) and anti–cyclic citrullinated peptide (anti‐CCP) titers (*P* < 0.0001). ERE expression was highest in circulating eRA B cells, particularly naïve B cells compared with ePsA, with possible ERE regulation by SAM and HD Domain Containing Deoxynucleoside Triphosphate Triphosphohydrolase 1 transcription (SAMDH1) implicated and associations with *IFNA* again observed. Finally, in established RA synovium, LTRs, particularly human endogenous retroviral sequence K (HERVK), were most increased in RA compared with OA, in which, for all synovial subsets (monocytes, B cells, T cells, and fibroblasts), ERE expression associated with increased IFN‐I signaling (*P* < 0.001).

**Conclusion:**

Peripheral blood and synovial ERE expression is examined for the first time in eRA, highlighting both a potential causal relationship between ERE and IFN‐I production and an intriguing association with anti‐CCP autoantibodies. This suggests EREs may contribute to RA pathophysiology with implications for future novel therapeutic strategies.

## INTRODUCTION

Type 1 interferons (IFN‐Is) have pleiotropic effects on the immune system and prime cellular responses to effectively clear, typically viral, infection.^1^ In this context, widespread cellular activation is desirable, but in the absence of infection, IFN‐I–associated increased cellular priming or activation can be inappropriate.^2^ Excess IFN‐α can promote a breach of tolerance in autoantibody producing B cells as well as facilitate more effective presentation of antigen, potentially of self‐components.^3^ We have previously demonstrated increased IFN‐I signaling and serum IFN‐α levels in early rheumatoid arthritis (eRA) with negative prognostic implications on initial disease control and clinical outcomes.^4^, ^5^ An elevated interferon gene signature (IGS) also increases the likelihood of progression to RA in at risk populations, such as those with anti–cyclic citrullinated peptide (anti‐CCP)–positive arthralgia.^6^, ^7^ However, it remains unknown what drives this IFN‐I release in eRA.

Endogenous retrotransposons or retroelements (EREs) are sequences of DNA derived from ancient transposable elements, such as retroviruses, that have been historically incorporated into the genome.^8^ Although the majority are inactive, some have retained transcriptional activity, and their replication cycle and organization is similar to exogenous retroviruses, such as HIV.^8^ EREs as a group can be subdivided into endogenous retroviruses (ERVs), often detected as long terminal repeats (LTRs), long interspersed nuclear element 1 (LINE‐1), and short interspersed nuclear elements (SINEs), most commonly “Alu.” Some EREs, such as ERVs, can replicate, generating a strand of messenger RNA (mRNA) and, subsequently, a double‐stranded RNA product, which then inserts into a unique region of the genome, often separate from the area of origin; depending on the site of insertion, this potentially disrupts protein coding regions.^8^, ^9^ This process of active retrotransposition results in the accumulation of cytosolic DNA, which triggers an interferon regulatory factor 3 (IRF3)–dependent innate immune response, including the release of IFN‐I.^10^, ^11^, ^12^ Indeed, single mutations in human genes that regulate retroelement replication, such as *TREX1* or *SAMHD1*, cause type 1 interferonopathies such as Aicardi‐Goutières syndrome (AGS).^13^

The potential for an association between EREs and IFN‐I production in autoimmunity is increasingly appreciated.^10^, ^11^, ^12^, ^13^, ^14^ In diseases in which IFN‐I are known to play a pathogenic role, such as systemic lupus erythematous or primary Sjögren's disease, there is evidence of increased LINE‐1 activity in disease relevant tissue associated with increased local IFN‐α production.^14^ Although established RA synovium was shown to overexpress LINE‐1 nearly two decades ago,^15^ interferon response gene profiles within the IGS vary between autoimmune diseases, potentially implicating disparate interferonogenic triggers.^16^ This highlights the need to examine for any association between EREs and IFN‐I in RA specifically. Furthermore, some ERVs retain their ability to produce viral protein, and ERV viral protein products have been detected in the peripheral circulation of patients with RA and linked to autoantibody generation.^17^, ^18^, ^19^, ^20^

To date, EREs have not been examined in RA in relation to IFN‐I production or in eRA, a period in which IFN‐I signaling, and autoantibody generation, is important.^4^, ^5^ We therefore explored ERE expression in whole‐blood, circulating lymphocyte subsets and synovial tissue samples from treatment‐naïve patients with eRA and hypothesized a potential association between ERE activity and (1) IFN‐I generation and/or (2) autoantibody generation.

## METHODS

### Patient cohorts

Glucocorticoid and disease‐modifying antirheumatic drug–naïve patients attending Newcastle upon Tyne Hospitals were enrolled for this study from the Northeast Early Arthritis Cohort at the point of diagnosis of either RA (with reference to 2010 American College of Rheumatology/EULAR RA classification criteria; patients with eRA) or psoriatic arthritis (early psoriatic arthritis [ePsA]), which constituted a non‐RA early inflammatory arthritis control group with the same disease duration.^21^ Contemporaneous clinical parameters were recorded, including disease activity scores (Disease Activity Score‐28 using the erythrocyte sedimentation rate [ESR]), Igs (IgG, IgA, and IgM), markers of inflammation (C reactive protein [CRP] and ESR), and serological status, (rheumatoid factor [RF] and anti‐CCP titers).

### IGS and serum cytokines

Serum samples were spun and frozen within four hours of blood draw, undergoing no more than one freeze‐thaw cycle before measurement of IFNγ, interleukin 6 (IL‐6), IL‐12p70, tumor necrosis factor α (TNFα), IL‐1β, IL‐2, IL‐13, IL‐4, and IL‐10 by MSD technology (Meso Scale Discovery) as per manufacturers’ instructions. Serum IFNα was measured using the digital Simoa platform as described.^4^ Serum IFN monoclonal antibodies (specific for all IFNα subtypes) were isolated from patients with autoimmune polyendocrinopathy–candidiasis–ectodermal dystrophy (APECED)^22^ and provided to author DD by Immunoqure under a material transfer agreement (MTA). The IGS was generated from whole‐blood RNA, as described previously, by the mean expression of five interferon response genes (IRGs) *MxA*, *IFI6*, *OAS1*, *ISG15*, and *IFI44L*.^5^

### Flow cytometric cell sorting

For all samples, peripheral blood mononuclear cells (PBMCs) were isolated from whole blood using density centrifugation and underwent immediate flow cytometric sorting. Plasmacytoid dendritic cells (pDCs), conventional CD1c^+^ DCs, CD4^+^ T cells, CD8^+^ T cells, CD19^+^ B cells, and CD14^+^ monocytes were sorted as previously described,^23^ and B cell subsets including naïve B cells (CD19^+^IgD^+^CD27^−^), memory B cells (CD19^+^IgD^−^CD27^+^), CD5^+^ B cells (CD19^+^CD5^+^), and age‐associated B cells (ABCs) (CD19^+^CD11c^+^CD21^−^) were flow cytometrically sorted from PBMCs from both patients with eRA and ePsA, as previously described.^24^

### Endogenous retroelement quantification

#### Whole‐blood and circulating lymphocytes

Whole‐blood RNA was isolated using the Tempus Spin Isolation Kit (Tempus, Thermo Fisher Scientific) and treated with TurboDNase (Ambion) to remove any contaminating genomic DNA (gDNA). The absence of gDNA was confirmed by *HBP1* polymerase chain reaction (PCR) and gel electrophoresis (Supplementary File 1). RNA was reverse‐transcribed to complementary DNA (cDNA) using Superscript II (Thermo Fisher Scientific) and gene specific primers for LINE‐1 (L1) and housekeeper TATA box binding protein (TBP) as previously described,^25^ (Supplementary File 2). Reverse transcription PCR (RT‐PCR) using SYBR Green Master Mix (Thermo Fisher Scientific) was performed using specific primers for L1‐5′ untranslated region (UTR) and *TBP* (Supplementary File 1). cDNA generation using L1‐specific primers close to 3′ end and quantitative PCR using primers targeting 5′UTR of L1 enhanced the detection of authentic full‐length L1 transcriptions. Consensus sequences from across L1 subtypes were used in the primers to maximize relevant transcription identification. Subsequent expression was displayed as a ratio of a biologic control (HEK293T cell line ERE expression) to minimize any batch effects.

Sorted cell subsets were processed as previously described.^23^, ^24^ In brief, the contemporaneous lymphocyte subsets had RNA isolated using Qiagen RNeasey Plus Micro Kits, which was then applied to a gDNA Eliminator spin column (both Qiagen) as per the manufacturer's instructions. For the B cell subsets, 15,000 cells were sorted into RF10 (RPMI 1640 culture medium containing 10% fetal calf serum; both Sigma‐Aldrich). After sorting, the cells were pelleted and lysed in RNeasy Lysis Buffer (Qiagen). Either 50 ng of RNA or the lysate from 15,000 cells, respectively, was loaded onto a NanoString nCounter Human immunology V2 Panel chip (NanoString Technologies Inc), including customized probes against SINE Alu element AluYa5, LTR5, and LINE‐1 5′UTR (L1‐5′UTR) (Supplementary File 3), and run according to manufacturer's instructions. Again, consensus sequences from these three ERE families were used in the probes based on the authors’ previous work,^25^ extended to meet NanoString capture probe criteria.

### Synovial tissue

Synovial biopsy specimens of wrist or knee joints were retrieved as described^26^ using a 16‐gauge Quick‐Core Biopsy Needle (Cook Medical) or Temno Biopsy Needle (Carefusion/Becton Dickinson) from consenting individuals before the commencement of immunomodulatory therapy, including systemic glucocorticoids. Tissue was paraffin‐embedded as previously described, approximately 24 hours after collection, into 10% neutral buffered formalin.^27^ Total RNA was extracted from curls taken from formalin‐fixed paraffin‐embedded (FFPE) blocks using the RNeasy FFPE kit and quality‐assessed by Qubit fluorometric quantitation according to the manufacturers’ instructions. Samples that passed quality control (25 ng) for transcriptional profiling employed the nCounter PanCancer Immune profiling codeset panel, modified to include the probes for EREs, as previously mentioned (Supplementary File 3).

### In silico analysis of established RA and osteoarthritis synovial tissue single‐cell sequencing data sets

*RepEnrich* is a computational method that allows for the analysis of repetitive elements in any organism with a reference genome available that has repetitive element annotation.^28^ This platform was applied to freely available established RA and osteoarthritis (OA) synovial tissue single‐cell data (https://immunogenomics.io/ampra) from the Accelerating Medicines Partnership Rheumatoid Arthritis and Systemic Lupus Erythematosus Consortium.^28^ Full analysis details are in Supplementary File 4, and cohort demographic data are available from the study by Zhang et al.^29^ Comparison between synovial cell subsets was performed using the cellular clustering described in the study by Zhang et al.^29^

### Statistical analysis

GraphPad Prism (V.5.0; GraphPad Software) and R Core Team (2020) software were used. Univariate generalized linear models, Mann‐Whitney U‐tests, one‐way analysis of variance (ANOVA) (with Tukey's post hoc analysis), and Wilcoxon‐signed rank tests were performed, employing a significance threshold in which α = 5%. Lymphocyte and B cell subsets NanoString nCounter data analysis was performed in R (v4.2.1), as described previously.^23^, ^24^ Synovium data were processed similarly, as outlined in Supplementary File 5.

### Data availability statement and ethics statement

The data are available for the purposes of academic research on reasonable request to the corresponding author. For the early disease data, all patients provided written, informed consent to participate in the study, which was approved by the Northeast – Newcastle and North Tyneside 2 Research Ethics Committee (12/NE/0251). For established RA and OA data, consent was obtained as previously outlined.^29^

## RESULTS

### Patient cohorts

The whole‐blood LINE‐1 analysis cohort included 56 patients with eRA. Simultaneous B cell, T cell, DC, and monocyte cell–specific retroelement expression was obtained from eight patients with rheumatoid factor‐positive and anti‐CCP–positive (double seropositive) eRA. B cell subset expression was assessed between double‐seropositive patients with eRA and ePsA (n = 4 each) matched for age and sex, with comparable levels of inflammation (CRP and ESR). The synovial tissue cohort comprised 22 patients with eRA. Full demographic and clinical data are shown for all the cohorts in Table 1.

**Table 1:** Demographic data of patients and controls*

Cohort	Whole blood	Circulating lymphocyte subsets	Circulating B cell subsets		Bulk synovial tissue
	eRA (n = 56)	eRA (n = 8)	eRA (n = 4)	ePsA (n = 4)	eRA (n = 22)
Age, median (range), yr	58 (30–87)	56 (49–64)	62 (63–78)	62 (60–80)	63 (41–78)
Sex ratio, M:F	1:1.8	3:1	1:1	1:1	1:1
Seropositive (either anti‐CCP or RF), n (%)	43 (77)	8 (100)	4 (100)	0	14 (64)
CRP, median (range)	8 (4–114)	7 (4–56)	9 (4–127)	7 (4–167)	22 (4–62)
DAS‐28‐ESR, median (range)	4.3 (1.3–7.6)	3.71 (1.63–6.18)	4.47 (1.33–8.53)	n/a	4.74 (2.47–7)

* Anti‐CCP, anti–citrullinated peptide; CRP, C‐reactive protein; DAS‐28‐ESR, Disease Activity Score‐28 using the erythrocyte sedimentation rate; ePsA, early psoriatic arthritis; eRA, early rheumatoid arthritis; F, female; M, male; n/a, not applicable; RF, rheumatoid factor.

### eRA synovial and peripheral blood endogenous retroelement expression and IFN‐α

**Figure 1:**
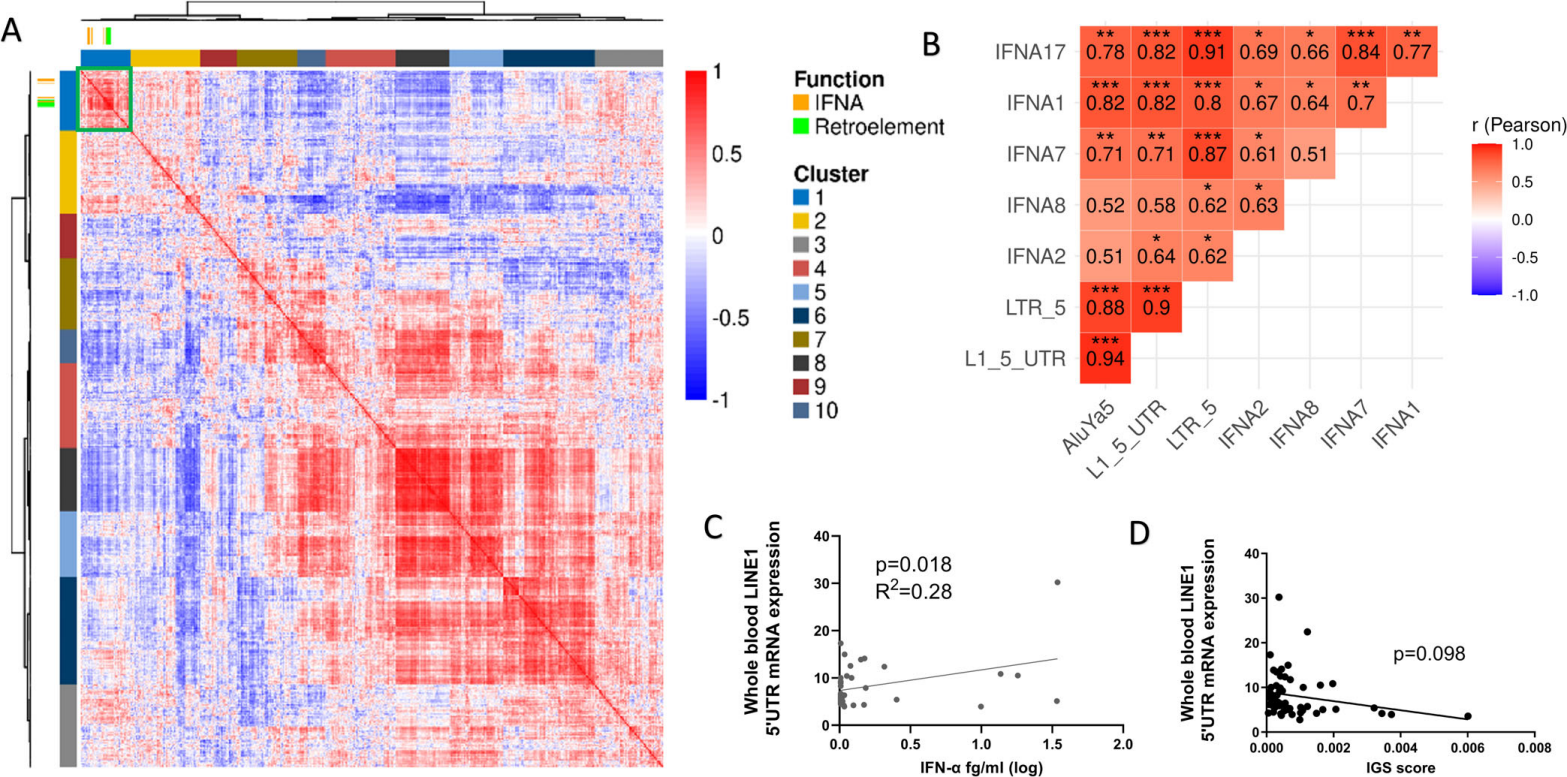
(A) Hierarchical clustering of early RA bulk synovial tissue gene expression correlations. Rows and columns depict genes, and the color bar represents the Pearson's coefficient (r) of their pairwise gene expression correlation. Dendrograms show hierarchical clustering of the genes by their expression correlation patterns. *IFNA* and EREs are highlighted by individual gene markers and the locations of their correlations by the green box. (B) Heatmap of correlation profiles between early RA bulk synovial *IFN* transcription and ERE classes. Significant (Benjamini Hochberg False Discovery Rate adjusted *P* value < 0.05) correlations are highlighted, *P < 0.05, **P < 0.01, ***P < 0.001. (C) Whole‐blood L1–5′UTR expression was analyzed in patients with early RA and shown in arbitrary units in relation to expression in HEK293T. Linear regression of early RA whole blood (L1‐5′UTR) and circulating IFN‐α protein level, n = 42, and (D) whole‐blood IGS, n = 56, is shown. AluYa5, Alu element Ya5; ERE, endogenous retroelement; IFN, interferon; IGS, interferon gene signature; L1–5′UTR; LINE‐1 5′UTR; LINE‐1, long interspersed nuclear element 1; LTR, long terminal repeat; mRNA, messenger RNA; RA, rheumatoid arthritis; UTR, untranslated region.

In eRA whole‐synovial tissue samples, hierarchical clustering of coexpression correlations of all available genes demonstrated clustering of *IFNA* and retroelements (Figure 1A). A heatmap of the correlations between genes within the ERE cluster is shown in Supplementary File 6. Pathway analysis (Kyoto Encyclopedia of Genes and Genomes [KEGG] pathway database) of the ERE cluster implicated enrichment of JAK‐STAT signaling (*P* = 0.004), primarily due to the association with IFN‐I, an enrichment also seen in gene ontology (GO) terms (*P* = 2.71 × 10^−6^) (Supplementary File 6). *IFNA* transcripts (*IFNA1*, *IFNA2*, *IFNA7*, *IFNA8*, and *IFNA17*) significantly positively associated with all classes of ERE (Figure 1B) but was strongest for LTR5: *IFNA17*, R = 0.91, Pearson correlation coefficient, Benjamini Hochberg False Discovery Rate (BH) adjusted *P* = 4.68 × 10^−9^. A similar significant association between ERE activity and *IFNA* was reported in patients who were either anti‐CCP positive (n = 13) or anti‐CCP negative (n = 22), for example, LTR5 and *IFNA17* were R = 0.92 vs 0.88, respectively.

There was no significant association with ERE expression and any other cytokine transcript including IFNγ, IL‐6, IL‐12 p70, TNFα, IL‐1β, IL‐2, IL‐13, IL‐4 and IL‐10 (data not shown). In eRA whole‐blood samples, there was a significant positive association between LINE‐1 transcript expression and circulating IFN‐α protein, *P* = 0.018 (Figure 1C). This was not seen with any of the other circulating cytokines measured: IFNγ, IL‐6, IL‐12 p70, TNFα, IL‐1β, IL‐2, IL‐13, IL‐4, and IL‐10, *P* > 0.05 for all, data not shown. There was no significant association between the whole‐blood IGS and LINE‐1 transcription expression (Figure 1D), despite a positive trend (*P* = 0.06) between the IGS and circulating IFN‐α (data not shown). Finally, whole‐blood LINE‐1 expression did not correlate with age or sex (Supplementary File 7).

### eRA whole‐blood LINE‐1 expression and correlation with anti‐CCPtiters

**Figure 2:**
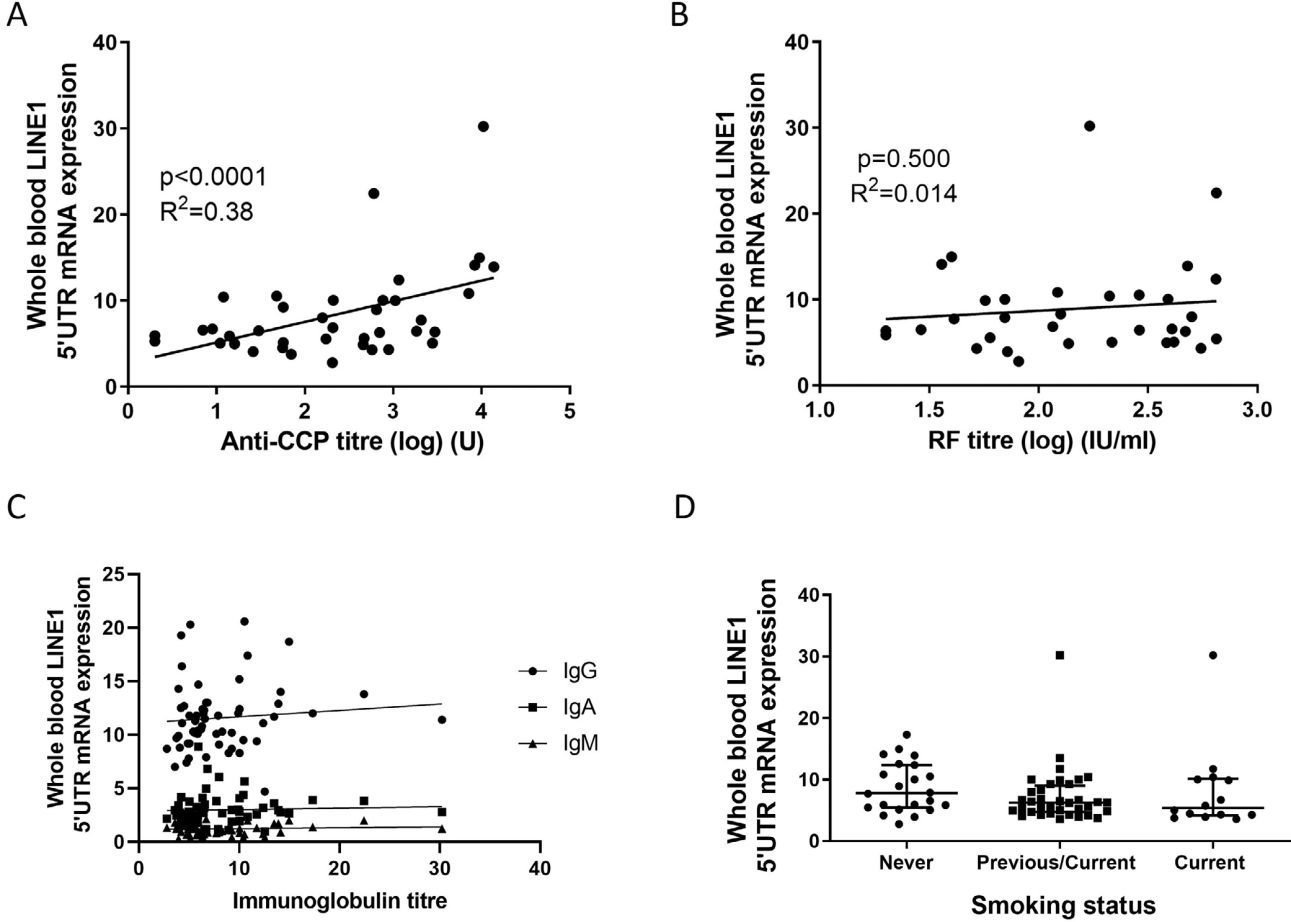
Whole‐blood L1–5′UTR expression was analyzed in patients with early RA (n = 56) and is shown in arbitrary units in relation to expression in HEK293T cells. (A and B) Linear regression between patients with early RA (n = 39) anti‐CCP titres or RF titres (n = 34) and whole‐blood L1–5′UTR expression. (C) Linear regression between L1‐5′UTR expression and circulating Igs (IgA, IgM, and IgG) in patients with early RA (n = 56), *P* > 0.05 for all. (D) Comparison of L1‐5′UTR expression between patients with early RA based on smoking status: never, previous/current, and current. Anti‐CCP, anti–cyclic citrullinated peptide; L1–5′UTR; LINE‐1 5′UTR; LINE‐1, long interspersed nuclear element 1; mRNA, messenger RNA; RA, rheumatoid arthritis; RF, rheumatoid factor.

There was a significant positive association between anti‐CCP titers (International Units [IU]) and LINE‐1 (L1‐5′UTR, linear regression, *P* < 0.0001, R^2^ = 0.38), which was not seen for RF titers (Figure 2A and B). Expression did not appear to reflect global B cell function because there was no association between circulating Ig levels IgM, IgG, or IgA and whole‐blood LINE‐1 (linear regression, *P* > 0.4 for all) (Figure 2C). Smoking is implicated in both anti‐CCP generation and ERE activity^30^, ^31^; however, there was no significant difference in whole‐blood LINE‐1 expression between cohorts based on smoking status (*P* > 0.05, ANOVA) (Figure 2D). There was also no significant association between LINE‐1 expression and disease activity (Disease Activity Score‐28), or its components including, tender joint count (TJC), swollen joint count (SJC), visual analogue scale (VAS), CRP, and ESR (data not shown).

### ERE expression in circulating eRA B cells, particularly naïve subsets, and associations with increased *IFNA* transcription

**Figure 3:**
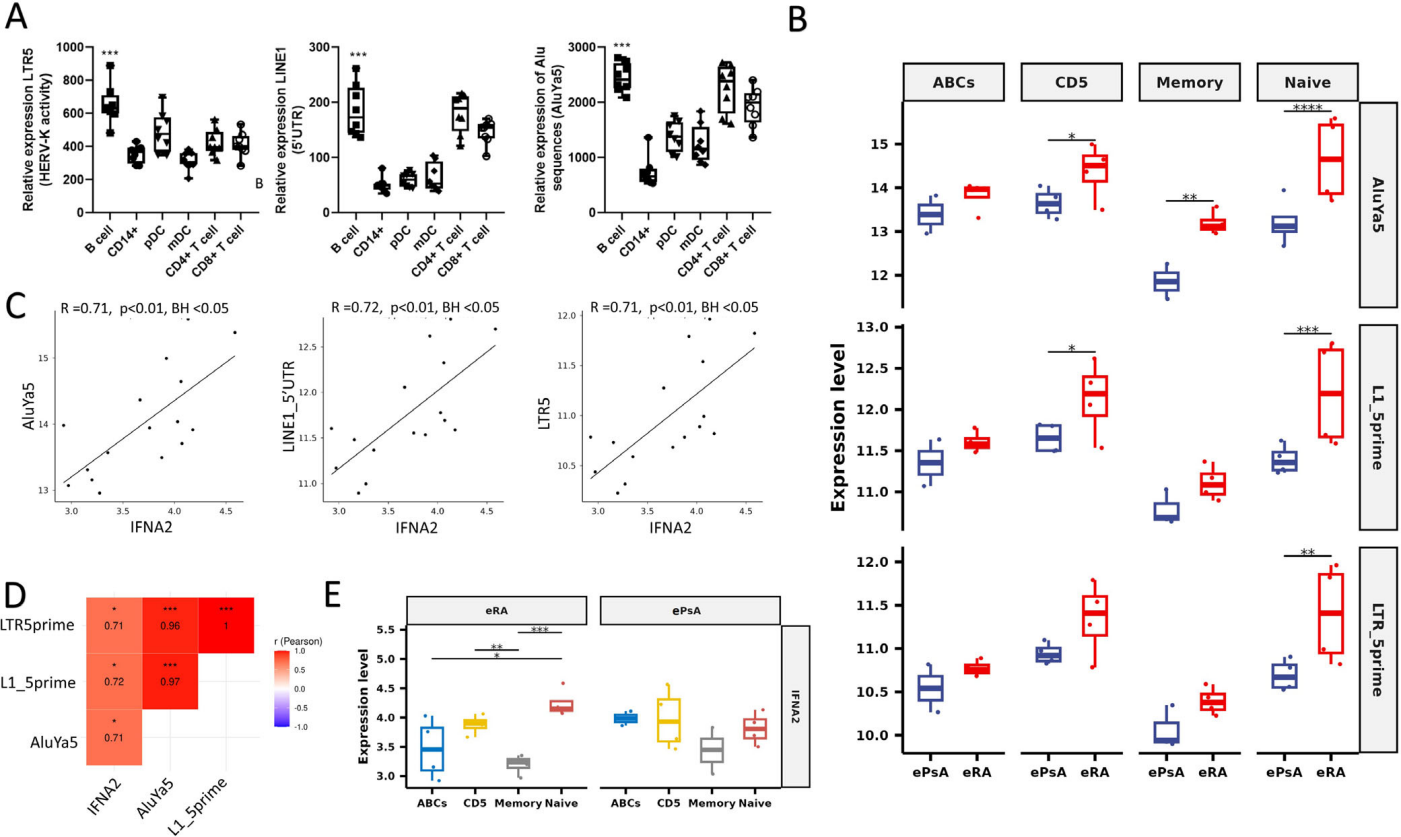
(A) Circulating lymphocyte (CD19^+^ B cells, CD14, pDCs, conventional mDCs, CD4, and CD8) retroelement (endogenous retroelements) expression (LTR5, LINE‐1 [L1‐5′UTR], AluYa5) in eRA (n = 8). Data are presented as box and whisker plots, in which the horizontal line represent the median value, the box represents upper and lower quartiles, and the whiskers represent ranges. Kruskal‐Wallis test, BH adjusted. (B) Expression of all retroelement classes in B cell subsets, age ABCs (CD19^+^CD11c^+^CD21^−^), CD5^+^ B cells (CD19^+^CD5^+^), memory B cells (CD19^+^IgD^−^CD27^+^), and naïve B cells (CD19^+^IgD^+^CD27^−^) from patients with eRA and early disease controls (ePsA), Wald test. (C) Pearson correlation coefficient of *IFNA2* and SINE (AluYa5), LINE‐1 (L1_5prime), and LTR5 (LTR_5prime) expression from eRA cells, *P* < 0.01, BH adjusted. (D) Heatmap of correlation profiles between *IFN* transcription and retroelement classes in pooled eRA B cell subsets. Significant (BH adjusted *P* value < 0.05) correlations are highlighted. (E) Comparison of *IFNA2* expression across B cell subsets in eRA and ePsA, paired t tests. *P < 0.05, **P < 0.01, ***P < 0.001. ABC, associated B cell; AluYa5, Alu element Ya5; BH, Benjamini Hochberg False Discovery Rate; CD4, CD4^+^ T cells; CD14, CD14^+^ monocyte; ePsA, early psoriatic arthritis; eRA, early rheumatoid arthritis; IFN, interferon; L1–5′UTR; LINE‐1 5′UTR; LINE‐1, long interspersed nuclear element 1; LTR, long terminal repeat; mDC, conventional CD1c^+^ DC; mRNA, messenger RNA; pDC, plasmacytoid dendritic cell; SINE, short interspersed nuclear element; UTR, untranslated region.

LTR5, LINE‐1, and AluYa5 expression was compared between lymphocyte subsets (B cells, pDCs, CD1c^+^ DCs, CD14^+^ monocytes, CD8^+^, and CD4^+^ T cells) from eight patients with double‐seropositive eRA. ERE expression was significantly increased in B cells compared with other lymphocyte subsets (Figure 3A). ERE activity in the B cell compartment was examined further comparing ABCs, naïve, memory and CD5^+^ B cells in eRA, with patients with ePsA as disease controls. There was a trend toward increased expression of ERE in patients with eRA in all subsets, which became highly significant for naïve B cells (Figure 3B).

When grouping together and pooling all the B cell subset transcriptomic data from our eRA cohort, ERE significantly associated with *IFNA* transcription (Figure 3C and D). Hierarchical clustering of coexpression correlations of all available genes further demonstrated clustering of *IFNA* and EREs (Supplementary File 8). Clusters were visually defined, and a heatmap of the correlations between the genes within the ERE cluster is shown in Supplementary File 9. Pathway analysis of this cluster in eRA alone demonstrated limited terms achieving significance; however, when examining pooled ePsA and eRA data, increased ERE expression was associated with enrichment of KEGG pathways relating to viral infection as well as phosphatidylinositol 3‐kinase/protein kinase B (PI3K/Akt) signaling, and GO terms were enriched for lymphocyte activation involved in immune response (*P* = 0.0005) (Supplementary File 9).

Furthermore, in eRA, there was significantly increased *IFNA2* transcript in naïve and CD5 B cells compared with memory B cells, a pattern not seen in ePsA (Figure 3E), and, when comparing directly between eRA and ePsA, there was a trend toward higher expression of *IFNA2* in eRA‐naïve B cells than in ePsA‐naïve B cells, although this was not significant (Supplementary File 10). Finally, to explore potential signaling pathways, we examined associations between EREs and key innate immune sensors, retinoic acid–inducible gene I (RIG‐I), melanoma differentiation‐associated protein 5 (MDA5), Toll‐like receptor 7 (TLR7), cyclic GMP‐AMP synthase (cGAS), and TLR9 in the pooled lymphocyte subsets. A significant positive association was only seen between RIG‐I and LTR5 (R^2^ = 0.64, *P* < 0.05) (Supplementary File 11).

### SAMHD1 is implicated in eRA peripheral blood B cell retroelement replication

**Figure 4:**
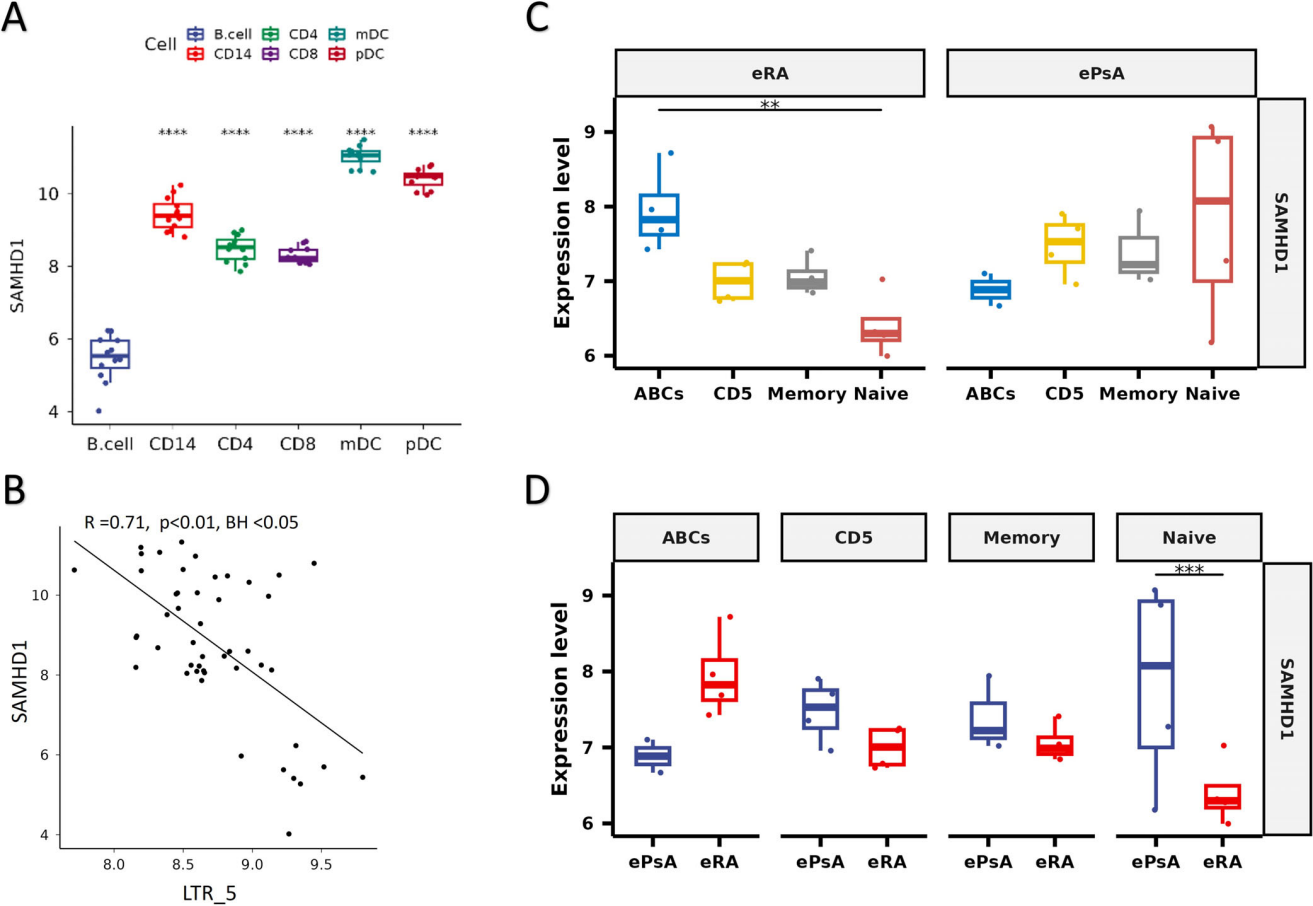
(A) Expression of *SAMHD1* as determined by Nanostring Technology in peripheral blood lymphocyte subsets (CD19^+^ B cells, CD14+ monocytes [CD14], CD4+ T‐cells [CD4], CD8+ T‐cells [CD8], conventional CD1c+ DCs [mDCs] and plasmacytoid dendritic cells [pDCs]) from patients with eRA (n = 8), with B cells used as a reference in pairwise paired t tests. (B) Pearson's correlation coefficient of *SAMHD1* and LTR5 in these same circulating lymphocytes grouped and pooled together, BH adjusted *P* < 0.0001. (C) *SAMHD1* expression, determined by Nanostring Technologies, was examined in age‐associated B cells (ABCs, CD19^+^CD11c^+^CD21^−^), CD5^+^ B cells (CD19^+^CD5^+^), memory B cells (CD19^+^IgD^−^CD27^+^), and naïve B cells (CD19^+^IgD^+^CD27^−^) from patients with eRA and patients with ePsA. Differences in *SAMHD1* expression was examined within disease cohort and (D) between disease cohorts. Wald test with BH adjusted and raw *P* values are shown for 3C and 3D, respectively. **P < 0.01, ***P < 0.001. Apparent discrepancies in values on Y columns between panels A, C, and D reflect technical variation in assay reference ranges. ABC, age associated B cell; BH, Benjamini Hochberg False Discovery Rate; CD4, CD4^+^ T cells; CD14, CD14^+^ monocyte; ePsA, early psoriatic arthritis; eRA, early rheumatoid arthritis; LTR, long terminal repeat; mDC, conventional CD1c^+^ DC; pDC, plasmacytoid dendritic cell; SAMHD1, SAM and HD Domain Containing Deoxynucleoside Triphosphate Triphosphohydrolase 1. Color figure can be viewed in the online issue, which is available at http://onlinelibrary.wiley.com/doi/10.1002/art.43083/abstract.

We examined expression of key enzymes involved in ERE activation in circulating B cells. SAM and HD Domain Containing Deoxynucleoside Triphosphate Triphosphohydrolase 1 (SAMHD1) transcript, an enzyme limiting retroelement replication,^32^ was significantly reduced in eRA B cells when compared with all other circulating lymphocytes (*P* < 0.001) (Figure 4A). Furthermore, eRA expression of SAMHD1 inversely correlated with ERE transcript expression examined across all lymphocyte subsets (Figure 4B and Supplementary File 12). SAMHD1 expression was significantly reduced (*P* < 0.01) in eRA‐naïve B cells compared to ABCs, and a trend was noted for reduced expression compared with memory and CD5^+^ B cells. This pattern was not seen in ePsA controls (Figure 4C). Finally, SAMDH1 was uniquely and significantly reduced in eRA‐naïve B cells (*P* < 0.005) compared with ePsA‐naïve B cells (Figure 4D). Ribonuclease H degrades RNA in RNA/DNA hybrids and expression of one of its key components, Ribonuclease H2 subunit A (RNASEH2A), was similar across all lymphocyte subsets and did not correlate with ERE expression. Conversely, three prime repair exonuclease 1 (TREX1), another key enzyme negatively regulating ERE expression, varied by cell subset, with the lowest expression being in T cells. There was an inverse association between TREX1 and ERE expression in eRA pooled lymphocytes, but expression in B cell subsets between eRA and ePsA cohorts was comparable (all in Supplementary File 13). Bulk synovial expression of ERE in the patients with eRA did not associate with *SAMHD1*, *TREX1*, or *RNAseH2* and, in neither circulating eRA lymphocyte subsets nor synovial tissue did *DNMT1*, *DNMT3A*, or *DNMT3B* (DNA methyltransferase enzymes, important in the epigenetic regulation of EREs) associate with ERE expression.

### ERE in RA synovial cell subsets and IFN‐I signaling

**Figure 5:**
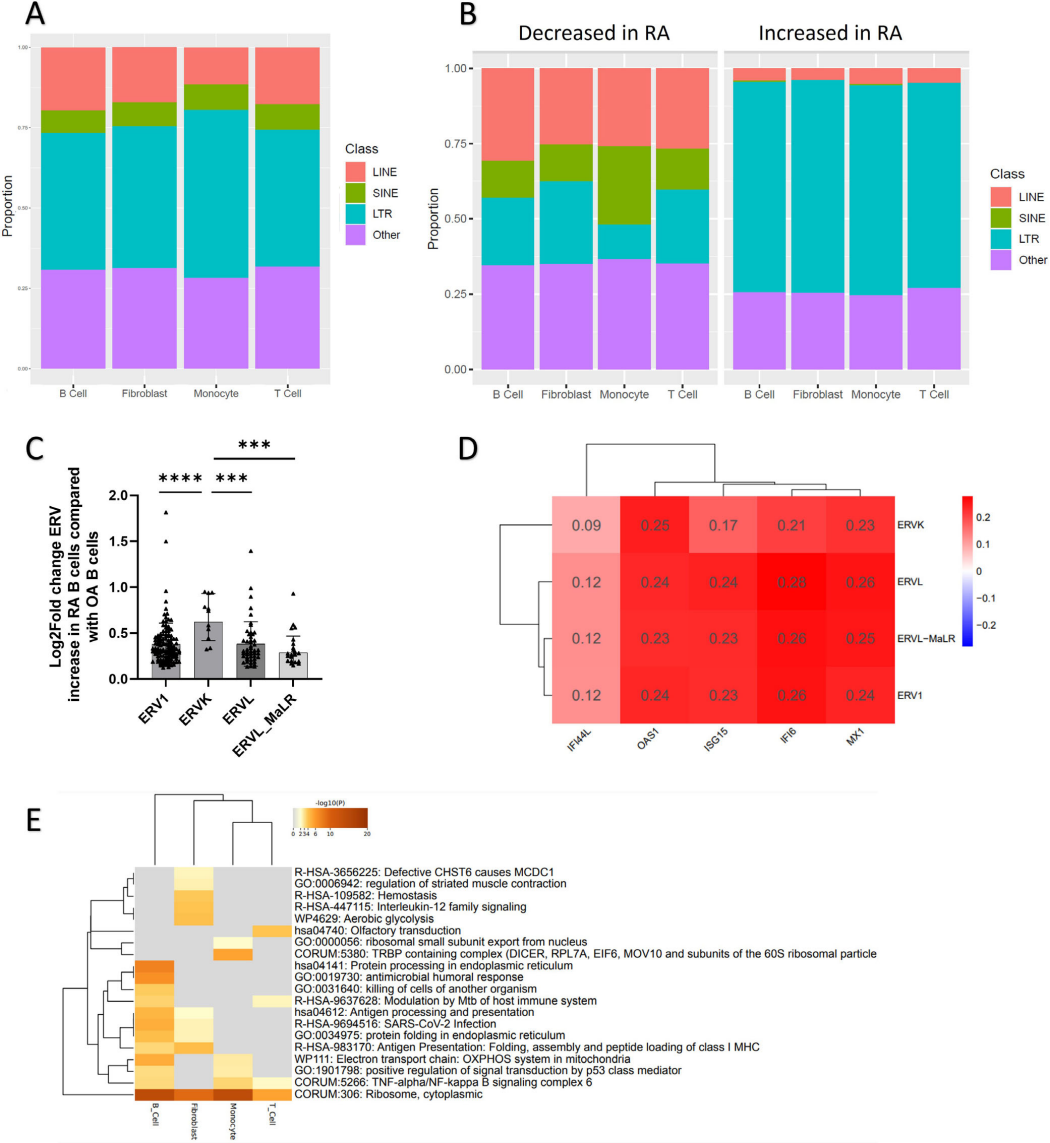
RepEnrich was applied to synovial scSeq data from established RA and OA controls, and repeat element enrichment/ERE were identified. Cellular clusters included monocytes, fibroblasts, T cells, and B cells. (A) The proportion of scSeq repeat element/ERE expression, grouped by ERE class, in each cell subset in established RA synovial tissue. (B) Proportion of individual EREs, grouped by class, with either increased or reduced expression in RA versus OA. (C) The LTR class is divided into ERV1, ERVK, ERV, and ERV_MaLR families, with individual ERE expression counts within each family. Depiction of increased differential expression (Log2FoldChange) in RA compared with OA of each individual ERE count within each ERV family. This is compared between the individual ERV families, Kruskall‐Wallis, BH adjusted *P* = 0.0006, and individual Mann‐Whitney U‐tests. (D) Hierarchical clustering of correlations between gene expression of interferon response genes and repeat element enrichment counts in RA B cells. Pearson's correlation coefficients depicted. All correlations were significant, BH *P* < 0.001. (E) MetaScape pathway analysis of all genes with a correlation of ≥0.4 with LTR repeat elements in each of the individual cell subsets. The top 20 pathways are depicted. ****P < 0.0001, ***P < 0.005. BH, Benjamini Hochberg False Discovery Rate; ERE, endogenous retroelements; ERV, endogenous retrovirus; LINE, long interspersed nuclear element; LTR, long terminal repeat; MaLR, mammalian‐apparent LTR retrotransposons; OA, osteoarthritis; RA, rheumatoid arthritis; scSeq, single‐cell RNA‐sequencing; SINE, short interspersed nuclear element.

Given differences in ERE expression observed in peripheral blood subsets, but strong correlation profiles between eRA bulk synovial *IFN* transcription and ERE classes (Figure 1), we wished to examine synovial tissue in more detail. Synovial single‐cell transcriptomic data from patients with established RA and OA was grouped into B cells, fibroblasts, monocytes, and T cells as previously described^29^ and reinterrogated for repeat element expression. This generated multiple individual ERE expression counts that could be grouped into classes, such as LTR. Examination of these individual ERE expression counts, when grouped into classes, demonstrated that in all RA synovial cell subsets, at a single‐cell level, LTR was proportionally the most highly expressed class (Figure 5A). Individual ERE expression counts were compared between OA and RA, and the proportion of all individual ERE expression counts within each class that were comparatively either reduced or increased in RA for each cell subset were demonstrated (Figure 5B and Supplementary File 14). In RA, the majority of ERE expression counts within the LTR class were increased for all synovial cellular subsets, whereas counts in the LINE and SINE classes were predominantly decreased. Individual ERE fold changes are shown in Supplementary Data S1.

Given that the LTRs were most widely increased in RA, we examined this class in more detail. LTRs consist of ERV families—ERV1, ERVK, ERVL, and )(ERVL‐ mammalian‐apparent LTR retrotransposons [MaLR])—and individual ERE counts within these family clusters were compared between RA and OA. Individual ERE expression counts increased in RA B cells were compared with OA B cells for each ERV family and is shown in Figure 5C. When comparing the comparative increased counts (Log2Fold) in RA versus OA among the ERV families, overall expression was greatest in the ERVK family (Kruskal‐Wallis test, BH adjusted *P* < 0.0006) (Figure 5C).

For all ERV families, hierarchical clustering of correlations between gene expression and ERE expression counts was performed. In all cell subsets, there was a positive correlation between IFN‐I response genes (*IFI44L*, *OAS1*, *IFI6*, *ISG15*, and *Mx1*) and LTR expression grouped by ERV family. All correlations met statistical significance with BH adjusted *P* < 0.001. Figure 5D depicts B cell correlations (see Supplementary File 15 for remaining synovial cell subsets). *IFNA* counts were too low for comparable analysis to be performed in all cellular subsets. Pathway analysis of the top 20 pathways corelating with LTR repeats across cell subsets demonstrated enrichment of viral response (SARS‐CoV‐2), antigen processing pathways, and antimicrobial humoral response in B cells (Figure 5E).

## DISCUSSION

We examined EREs for the first time in drug‐naïve eRA and demonstrated that EREs are transcriptionally active in both whole‐blood and synovial tissue samples, with variable expression across circulating lymphocyte subsets. Expression was the highest in B cells, particularly naïve B cells, which was not seen in ePsA. We demonstrate for the first time in RA that ERE activity in blood and synovial tissue associates with increased IFN‐α at both the transcription and protein levels. We also saw a positive association between ERE and CCP titers, which was absent for RF. Cumulatively, these data offer intriguing insights into a potential role for EREs in RA pathophysiology.

In bulk synovial tissue samples, from both patients with seropositive and seronegative eRA, we identified a significant positive association between *IFNA* transcription and ERE expression, particularly noted with LTR5. LTR5 expression denotes ERVK (HML‐2, Human MMTV‐like, group 2) activity,^33^ which has been recently integrated into the human genome, and multiple copies possess potential biologic activity.^34^ Indeed, some of the most compelling evidence of the involvement of EREs in RA has implicated this ERV. It has been detected in the plasma of patients with RA, with higher levels associating with active disease.^35^ Although ERVK transcripts have previously been detected in established RA blood and synovial tissue samples,^35^, ^36^ this is the first time they have been demonstrated in early disease and in association with IFN‐α upregulation. Furthermore, when reanalyzing an independent, established RA synovial tissue single‐cell RNA‐sequencing (scSeq) data set in the public domain, we observed ERVK to be the most up‐regulated subtype when compared with other ERVs. In contrast to eRA bulk synovial tissue analyses, we could find no direct association between ERE expression and *IFNA* transcript in scSeq data from major cellular subsets, including B cells, fibroblasts, monocytes, and T cells, in established RA, although there was evidence of increased downstream IFN‐I signaling. We previously showed circulating IFN‐α declines during the first 6 months after RA diagnosis,^4^ potentially explaining this difference from eRA, although the influence of distinct cellular composition and/or sampling technique cannot be excluded. ERVs also induce TNFα,^37^ which induces IRG expression independently of IFN‐I in RA synovial fibroblasts,^38^ an association we also identified in our pathway analysis, and this process may become dominant in established disease. Nonetheless, the clear association of ERE expression in RA synovial tissue with both *IFNA* transcription and IFN‐I signaling mirrors what is seen elsewhere in systemic autoimmunity,^14^ as well as organ‐specific autoimmunity, such as type 1 diabetes, and warrants further exploration.

Circulating immune cell subsets and their activation may contribute to, or reflect, tissue‐specific processes. Notably, eRA synovial fluid IFN‐α levels are comparable to those in the circulation.^4^ In eRA, we demonstrated positive associations between EREs and circulating B cell *IFNA* transcript levels as well as between whole‐blood *LINE1* activity and circulating IFN‐α protein levels, the latter, again, in both seropositive and seronegative patients. The double‐stranded RNA sensor RIG‐I was potentially implicated in ERE sensing by these data, consistent with our previously reported association between RIG‐I and circulating IFN‐α levels in eRA.^4^ An association between *IFNA* transcription and whole‐blood retrotransposon activity has recently been reported in other autoimmune diseases,^39^ but our demonstration of an association with IFN‐α protein reinforces potential biologic relevance in RA. Similar to other autoimmune diseases,^39^ we did not find a significant association between ERE activity and downstream IFN‐I signaling, although we noted a trend toward an inverse association with the IGS. This apparent discrepancy may arise because many IRGs are potent LINE‐1–negative regulators.^40^ Thus, examining upstream IFN‐α protein levels, as we did here, may be optimal when delineating associations between IFN‐α and EREs.

This is the first time all major classes of EREs have been simultaneously examined in circulating lymphocyte subsets, wherein we found the highest ERE expression in B cells, particularly naïve B cells. Although background inflammation levels could affect ERE activity,^20^ expression levels were increased in RA B cell subsets compared with patients with ePsA matched for inflammation. Furthermore, ERE expression in eRA whole‐blood samples, although associated with circulating IFN‐α levels, was independent of other circulating inflammatory cytokines. In keeping with this differential ERE expression, there was increased *IFNA* transcription in RA‐naïve B cells. Single‐cell analysis of RA circulating B cell subsets previously demonstrated increased sensitivity to IFN‐α and increased *IFNA* transcripts in RA‐naïve B cells, resulting in increased basal activation and proliferation.^41^ The role of IFN‐α in B cell function and the pathophysiology of autoimmunity has been well established, whereby it can enhance B cell proliferation, activation, and autoantibody production.^3^ Pretreatment with IFN‐α also enhances pathologic B cell proliferative responses and plasmablast differentiation.^42^ This potentially associates EREs to known RA pathophysiologic processes via enhanced IFN‐I signaling.

Indeed, EREs may also be implicated in B cell–driven autoimmunity independently of IFN‐α, via antibody responses to cell components associated with ERE nucleic acid, allowing molecular mimicry and cross activation to occur.^17^, ^43^ Overlap between rheumatic disease–associated autoantibodies, including anti‐Ro60 and RF, have been linked to ERE activity.^19^, ^20^, ^44^ We also showed a significant positive association between whole‐blood LINE‐1 activity and anti‐CCP titers, but not RF. Pathway analysis suggested a positive correlation between synovial B cell EREs and antigen processing and presentation. Antibodies against human ERVK *env*, as well as against its citrullinated form, have been detected in established RA, are increased in anti–citrullinated protein antibody (ACPA)–positive patients, and positively correlate with anti‐CCP titers.^45^ These data suggest EREs may contribute to citrullinated antigen detected by ACPA and cumulatively hint at a role for EREs in promoting autoantibody generation in RA.

Variation in circulating B cell ERE expression was associated with a reciprocal decrease in *SAMHD1*. This enzyme depletes intracellular deoxynucleoside triphosphate (dNTP) pools, thus limiting ERE replication, and its deficiency has been implicated in interferonopathies.^46^ SAMHD1 expression can vary between cell subsets,^47^ with lower levels previously reported in B cells.^48^ In B cells, *SAMHD1* is increased in G1 cell cycle phases, wherein it can enhance the development of high affinity antibodies.^49^ Naïve B cells, in phase G0, theoretically therefore would have lower levels of SAMHD1 and thus increased ERE expression, as we demonstrated. We also saw enrichment of PI3K/Akt signaling in B cells, and in AGS, this pathway has been implicated in linking SAMHD1 deficiency to increased IFN‐I response.^50^ *SAMHD1* is classed as an IFN response gene, and the reduced expression in naïve B cells may appear paradoxical given the increased IFN‐α reported. However, in some primary human cells, *SAMHD1* levels did not change following IFN‐α exposure and, in reality, this relationship is likely to be more nuanced.^47^, ^51^ Finally, *SAMHD1* expression levels do not necessarily correlate with its deoxynucleoside triphosphohydrolase (dNTPase) activity and cellular dNTP availability^47^; nevertheless, the reciprocal variability in expression levels between ERE expression and *SAMHD1* are suggestive that an association may exist.

There are recognized differences among gene expression changes, pathways, upstream regulators, and cellular functional states between synovium and peripheral blood in RA.^52^ Indeed, we did not see any link between *SAMHD1* expression and synovial ERE expression. We hypothesize that other retrotransposon regulatory mechanisms may be more relevant in synovial tissue, such as epigenetic silencing,^14^, ^53^ with promoter methylation having been shown to affect LINE1 activity in autoimmune diseases.^14^, ^39^ Although we did not explore these mechanisms in detail, we did not see any difference between retrotransposon activity and DNA methyltransferase 1 (DNMT1), DNMT3A, or DNMT3B expression, known epigenetic modifiers of ERE activity.^39^ This may reflect the sample size but may also suggest other mechanisms are dominant, such as SAMHD1 in B cells, or even differential expression in the recently described human silencing hub (HUSH) complex, a known gatekeeper of ERE‐induced IFN‐I expression.^53^ These provide promising avenues for future research.

Study limitations include the theoretical nonspecific detection of EREs present within other transcripts. However, primer design and cDNA generation were optimized to detect full‐length transcriptions to minimize this possibility. For some analyses, particularly those relating to B cell subsets, patient numbers were limited, with large variation reported. This likely reflects the heterogeneity inherent in RA populations, and future studies focusing primarily on cellular subsets of interest, such as B cells, will allow analysis of larger cohorts and inclusion of seronegative patients, an aspect currently lacking in our work. Finally, longitudinal studies will also help inform any differences between early and established RA and how ERE activity may change with time or in response to treatment.

In conclusion, we examine for the first time ERE activity in eRA and present potentially important associations between ERE activity and IFN‐I, B cell function, and autoantibody generation. Within this context, it is intriguing that antiretroviral drugs highly active antiretroviral therapy (HAART) have ameliorated symptoms in RA.^54^ Further work is needed to comprehensively explore the putative pathogenic involvement of EREs in eRA. This will allow greater understanding of RA pathophysiology and potentially provide new therapeutic targets.

## AUTHOR CONTRIBUTIONS

All authors contributed to at least one of the following manuscript preparation roles: conceptualization AND/OR methodology, software, investigation, formal analysis, data curation, visualization, and validation AND drafting or reviewing/editing the final draft. As corresponding author, Dr Cooles confirms that all authors have provided the final approval of the version to be published, and takes responsibility for the affirmations regarding article submission (eg, not under consideration by another journal), the integrity of the data presented, and the statements regarding compliance with institutional review board/Declaration of Helsinki requirements.

## Supporting information


Disclosure form

**Appendix S1:** Supplementary Information

Data S1:
